# Artificial intelligence‐assisted colonoscopy: A prospective, multicenter, randomized controlled trial of polyp detection

**DOI:** 10.1002/cam4.4261

**Published:** 2021-09-03

**Authors:** Lei Xu, Xinjue He, Jianbo Zhou, Jie Zhang, Xinli Mao, Guoliang Ye, Qiang Chen, Feng Xu, Jianzhong Sang, Jun Wang, Yong Ding, Youming Li, Chaohui Yu

**Affiliations:** ^1^ Department of Gastroenterology Ningbo Hospital of Zhejiang University Ningbo China; ^2^ Department of Gastroenterology The First Affiliated Hospital, College of Medicine, Zhejiang University Hangzhou China; ^3^ Department of Gastroenterology Yuyao People’s Hospital of Zhejiang Province Yuyao China; ^4^ Department of Gastroenterology Taizhou Hospital of Zhejiang Province Linhai China; ^5^ Department of Gastroenterology The Affiliated Hospital of Medical School of Ningbo University Ningbo China; ^6^ Department of Gastroenterology Sanmen People’s Hospital Taizhou China; ^7^ Department of Gastroenterology Ningbo Yinzhou People’s Hospital Ningbo China

**Keywords:** artificial intelligence, cancer prevention, colorectal polyps, endoscopy, image analysis

## Abstract

**Background:**

Artificial intelligence (AI) assistance has been considered as a promising way to improve colonoscopic polyp detection, but there are limited prospective studies on real‐time use of AI systems.

**Methods:**

We conducted a prospective, multicenter, randomized controlled trial of patients undergoing colonoscopy at six centers. Eligible patients were randomly assigned to conventional colonoscopy (control group) or AI‐assisted colonoscopy (AI group). AI assistance was our newly developed AI system for real‐time colonoscopic polyp detection. Primary outcome is polyp detection rate (PDR). Secondary outcomes include polyps per positive patient (PPP), polyps per colonoscopy (PPC), and non‐first polyps per colonoscopy (PPC‐Plus).

**Results:**

A total of 2352 patients were included in the final analysis. Compared with the control, AI group did not show significant increment in PDR (38.8% vs. 36.2%, *p* = 0.183), but its PPC‐Plus was significantly higher (0.5 vs. 0.4, *p* < 0.05). In addition, AI group detected more diminutive polyps (76.0% vs. 68.8%, *p* < 0.01) and flat polyps (5.9% vs. 3.3%, *p* < 0.05). The effects varied somewhat between centers. In further logistic regression analysis, AI assistance independently contributed to the increment of PDR, and the impact was more pronounced for male endoscopists, shorter insertion time but longer withdrawal time, and elderly patients with larger waist circumference.

**Conclusion:**

The intervention of AI plays a limited role in overall polyp detection, but increases detection of easily missed polyps; ChiCTR.org.cn number, ChiCTR1800015607.

## Graphical abstract

Artificial intelligence (AI) assistance plays a limited role in overall polyp detection. AI system increases detection of easily missed polyps, including non‐first polyps, diminutive and flat polyps. The impact is greater for male operators, longer withdrawal time, and elderly obese patients.

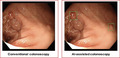

## 1 INTRODUCTION

Colorectal cancer (CRC) is a common and lethal disease worldwide. In China, CRC incidence and related deaths rank fifth among all cancers.^1^ Colonoscopy is an effective strategy to prevent CRC by early detection and removal of precancerous polyps.^2^ It has been showed that colonoscopic polypectomy can significantly reduce the incidence and mortality of CRC.^3^

However, as the quality of colonoscopy depends on the endoscopist, procedure‐related factors, and the patient/lesion itself, there is a risk of missing polyps.^4^, ^5^ The detection rate of polyps (especially adenomas) is negatively correlated with the CRC incidence and death.^6^, ^7^ Moreover, adenoma detection rate (ADR) is regarded as the main quality indicator of colonoscopy. However, in clinical practice, it is difficult to perform biopsy on every polyp during colonoscopy. In this case, polyp detection rate (PDR), as a simpler quality indicator of colonoscopy, has been proven to be a good proxy for ADR.^8^, ^9^ Therefore, we propose the need to improve PDR of colonoscopy.

Recently, artificial intelligence (AI)‐assisted polyp detection has gradually developed.^10^ There were a few prospective, single‐center, randomized controlled trials (RCTs) on real‐time polyp detection,^11^, ^12^, ^13^, ^14^, ^15^ showing that AI‐assisted colonoscopy had better performance than conventional colonoscopy in PDR and ADR. However, there are few multicenter RCTs for AI‐assisted polyp detection.

In previous research, we newly developed an AI‐assisted polyp detection system and used a large number of colonoscopy videos for system learning and testing. The pooled area under curve of the system was 0.939 with a sensitivity of 81.9% and a specificity of 98.4%. It can detect diminutive polyps, even polyps with incomplete imaging. Compared with other systems, its real‐time detection speed is faster at 25.8 ms per frame.^11^, ^12^, ^14^ In addition, we have used this system for offline analysis of colonoscopy videos in a large‐sample comparative study, finding that the system greatly improves polyp detection, thereby supporting its online use.

Here, we carry out a prospective multicenter RCT to evaluate the effectiveness of our AI‐assisted polyp detection system during real‐time colonoscopy.

## 2 METHODS

### 2.1 Study design and patients

In this prospective multicenter RCT, we screened patients who were scheduled for screening, surveillance, and diagnostic colonoscopy at six centers (ZJU Ningbo, Yuyao, Taizhou, NBU Ningbo, Sanmen, and Yinzhou) between April 2018 and March 2019, and randomized eligible patients to conventional colonoscopy (control group) or AI‐assisted colonoscopy (AI group). The results of real‐time polyp detection between two groups were collected to evaluate the effect of AI assistance.

Patients who met the following inclusion criteria, but not exclusion criteria, were eligible for the study. Inclusion criteria include: (1) patients with indications for screening, surveillance, or diagnostic colonoscopy; (2) the patient has undergone standard bowel preparation before colonoscopy; and (3) the patient has signed an informed consent form. Exclusion criteria include: (1) patients who were unwilling to participate in the study; (2) emergency colonoscopy; (3) colonoscopic polypectomy; (4) patients with a history of colorectal polyps; (5) patients with a history of colorectal resection; (6) patients with severe intestinal diseases, such as inflammatory bowel disease, colorectal cancer, intestinal obstruction, polyposis, etc.; and (7) patients with contraindications to colonoscopy.

By using random numbers generated by an investigator who was not involved in data collection and analysis, all eligible patients were randomly allocated (1:1) to conventional colonoscopy or AI‐assisted colonoscopy. Finally, the patients with qualified colonoscopy were included in the statistical analysis, that is, Boston bowel preparation scale (BBPS)^16^ score ≥ 6, cecal intubation, and withdrawal time (excluding biopsy time) ≥6 min.^17^ The study protocol was approved by Research Ethics Committee of the First Affiliated Hospital, College of Medicine, Zhejiang University (IRB No. 2018‐524) in accordance with Declaration of Helsinki. Patient consent for participation was obtained. All authors had access to the study data and approved the final manuscript.

### 2.2 Conventional colonoscopy versus AI‐assisted colonoscopy

**FIGURE 1:**
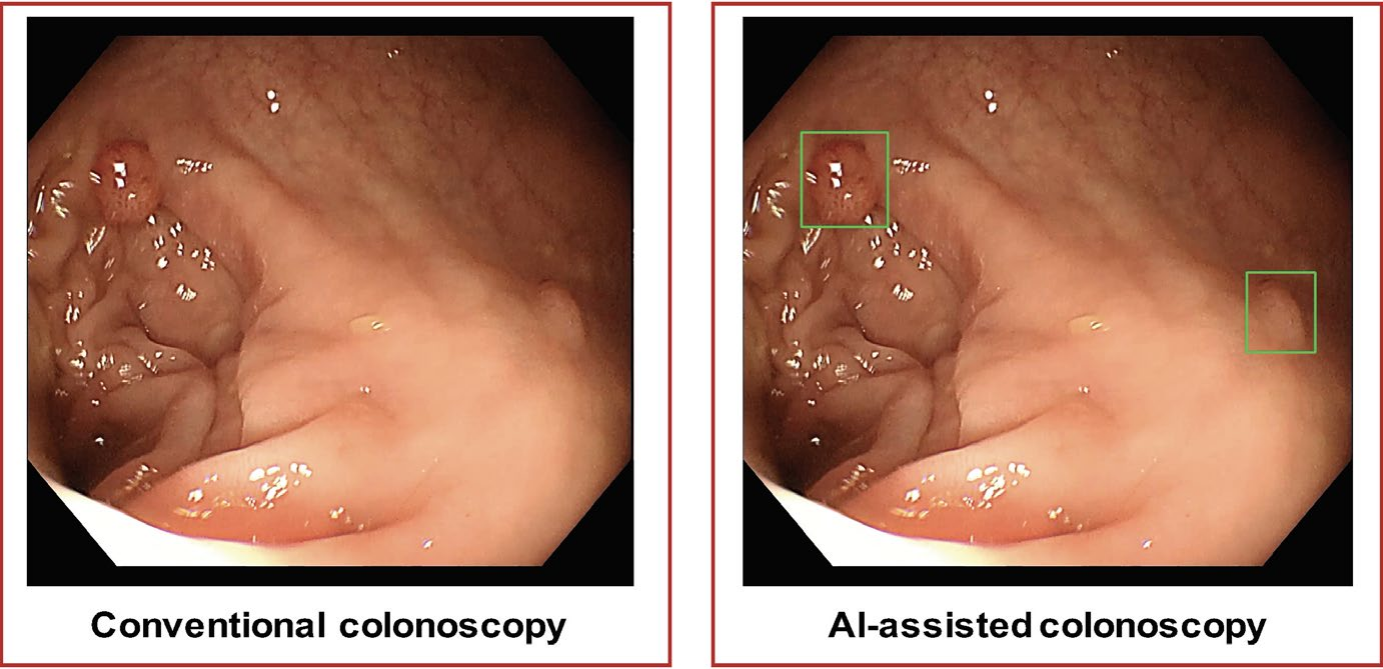
Diagram of study design. Eligible patients were randomly assigned to two groups. In control group, patients underwent a conventional colonoscopy with the AI‐assisted polyp detection system turned off; In AI group, patients underwent a colonoscopy with the AI system running, alerting the endoscopist to the detected polyp by a green indicator box, as well as a “Di” sound. AI, artificial intelligence

Colonoscopy was performed by a gastroenterology physician at the six centers using an identical colonoscope equipment (OLYMPUS CV‐290SL) with white‐light mode. Before the actual execution of the study, the technicians of Hithink RoyalFlush Information Network Co., Ltd. provided AI system training for endoscopists. Each endoscopist should perform at least three complete AI‐assisted colonoscopies under the supervision of a technician to ensure that they master the method of real‐time polyp detection using the AI system. During the formal study period, whether in the conventional arm or in the AI‐assisted arm, only the endoscopist was involved in polyp detection. For patients in control group, they underwent a conventional colonoscopy with the AI‐assisted polyp detection system turned off; for patients in AI group, they underwent a colonoscopy with the AI system running, alerting the endoscopist in real time to detected polyps visually (with a green indicator box, as shown in Figure 1) and audibly (with a “Di” sound).

Our AI‐assisted polyp detection system was developed using a deep learning method, based on the RestinaNet network model with feature pyramid network, ResNet‐50, classification and regression submodels, and focal loss. It combines the high‐level features with low‐level features extracted from the image to realize the detection of small polyps and even incomplete imaged polyps; by reducing the weight of easy‐to‐classify samples, the model pays more attention to hard‐to‐classify samples, thereby improving the classification ability; finally, the regression of the detection target gives the exact position of the polyp with a green indicator box on the screen. Through learning and testing of a total of 117,048 white‐light colonoscopy video frames from six centers, the sensitivity and specificity of the system reached 81.9% and 98.4%, respectively. At present, on a GeForce GTX 1080 Ti, the detection time of a single video frame is about 25.8 ms, which is faster than most previous AI systems. Furthermore, the system analyzes every third video frame, realizing real‐time detection.

### 2.3 Outcome measures

Similar to the outcome measures in previous studies,^18^ the primary outcome used in our study is PDR. The secondary outcomes include polyps per positive patient (PPP), which refers to the number of “positive patients” (patients with at least one polyp detected during colonoscopy) divided by the total number of patients, polyps per colonoscopy (PPC), calculated as the number of polyps detected during colonoscopy divided by the number of colonoscopies, and non‐first polyps per colonoscopy (PPC‐Plus), which means the number of “non‐first polyps” (polyps detected after the first one during colonoscopy) divided by the number of colonoscopies. The formulas are as follows:

$$\text{PDR}\;=\frac{\text{Number of positive patients}}{\text{Number of total patients}}\times 100\%$$

$$\text{PPP}\;=\frac{\text{Number of total polyps}}{\text{Number of positive patients}}$$

$$\text{PPC}\;=\frac{\text{Number of total polyps}}{\text{Number of colonoscopies}}$$

$$\text{PPC - Plus =}\;\frac{\text{Number of non - first polyps}}{\text{Number of colonoscopies}}$$

In addition, the endoscopist recorded the characteristics of the detected polyps:

(1) Polyp position: right colon (cecum, ascending colon), transverse colon (including liver curvature and spleen curvature), and left colon (descending colon, sigmoid colon, rectum).
(2) Polyp size: diminutive polyps (1–5 mm), small polyps (6–9 mm), and large polyps (≥10 mm). The size of the polyp was measured by placing an open biopsy forceps near it.
(3) Polyp morphology: polypoid (I), including protruded and pedunculated (Ip), protruded and subpedunculated (Isp), and protruded and sessile (Is); non‐polypoid (II), including slightly elevated (IIa), flat (IIb), and slightly depressed (IIc), based on the Paris classification scheme.
(4) Polyp histology (if necessary): adenomatous polyps, hyperplastic polyps, inflammatory polyps, and hamartomatous polyps, possibly with canceration.

### 2.4 Statistical analysis

For 80% power to detect a 5% difference (25% vs. 20%) in PDR at a two‐sided *α* level of 0.05 in a two‐group χ^2^ test, a sample size of 2178 was finally required. We planned an initial enrollment of approximately 2500 patients to allow subsequent exclusion.

We conducted the per‐protocol analysis for the study results using SPSS 20.0 software (IBM, Inc.). The data were expressed as mean ± standard deviation or number (percentage) as appropriate. The PDR of control group and AI group were compared by the χ^2^ test, and the PPP, PPC, and PPC‐Plus of the two groups were compared using the rank sum test. Logistic regression analysis was performed on factors that may affect AI‐assisted polyp detection. We selected these factors based on previous studies^19^, ^20^, ^21^, ^22^, ^23^, ^24^, ^25^ and evaluated them as effect modifiers. A *p* value of <0.05 was considered statistically significant.

## 3 RESULTS

**FIGURE 2:**
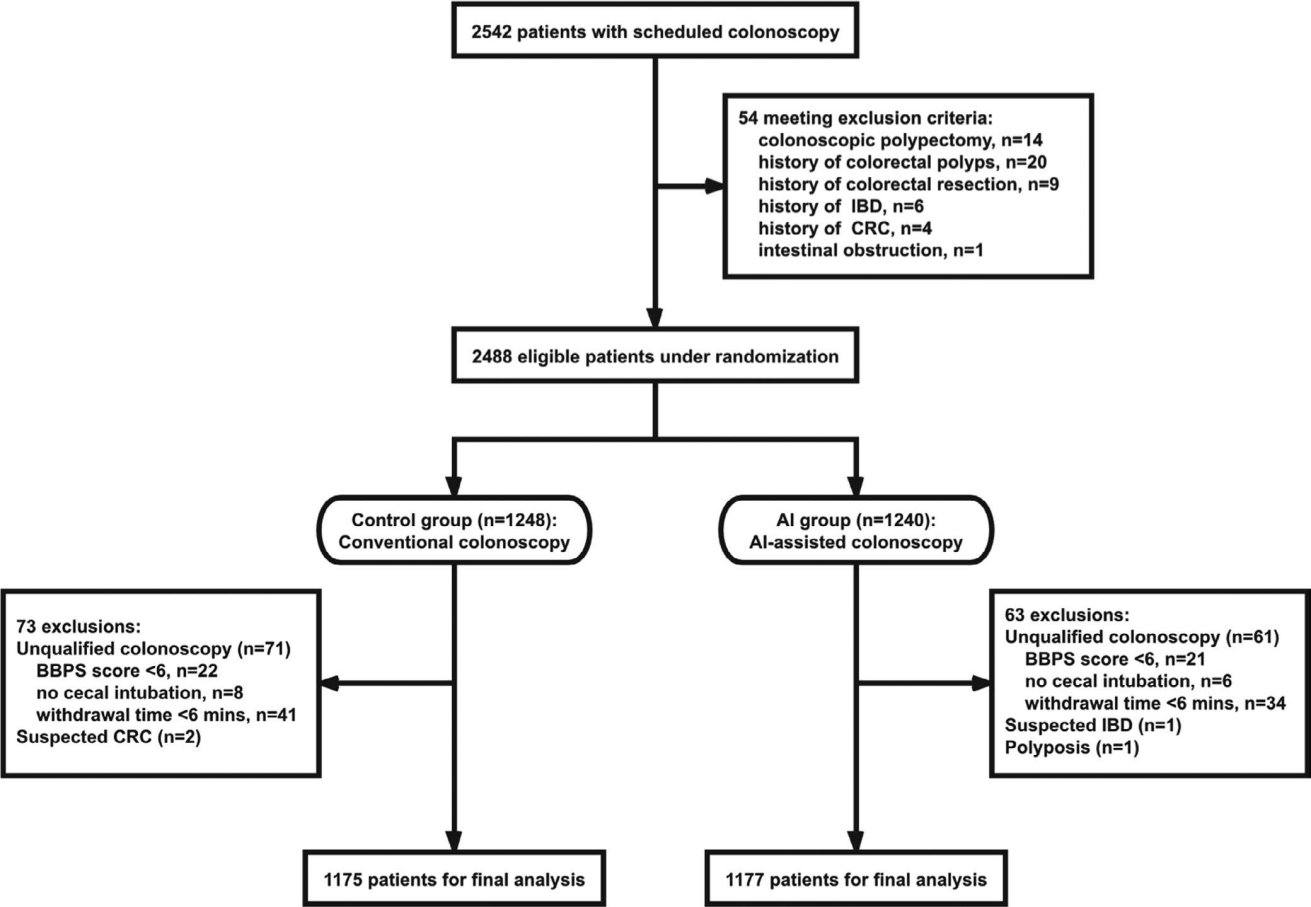
The study flowchart. (1) Screen patients based on inclusion and exclusion criteria. (2) 2488 eligible patients were randomized to the control or AI groups. (3) 1248 patients in control group underwent a conventional colonoscopy, while 1240 patients in AI group underwent a colonoscopy with real‐time use of AI‐assisted polyp detection system. (4) Excluding 136 patients with unqualified colonoscopy or suspected severe intestinal disease, the final statistical analysis was performed. AI, artificial intelligence; CRC, colorectal cancer; IBD, inflammatory bowel disease

A total of 2542 patients who underwent colonoscopy in the six centers from April 2018 to March 2019 were screened. Fifty‐six patients were excluded due to not meeting the eligibility criteria, and 2488 patients were randomly assigned to two groups, 1248 in control group and 1240 in AI group. After excluding 136 patients with unqualified colonoscopy or suspected severe intestinal disease (73 in control group and 63 in AI group), the final analysis was performed on 2352 patients, including 1175 in control group and 1177 in AI group (Figure 2). No adverse events related to system use occurred during the study.

The general characteristics of the two arms are shown in Table 1. There were no significant differences in the endoscopist characteristics (endoscopist experience and gender) and the procedure‐related factors (anesthesia, BBPS score, insertion, and withdrawal time) between the two groups. Regarding the patient characteristics, there were no significant differences in gender, age, body mass index (BMI), and waist circumference between the two groups. The study population was gender‐balanced, with an average age of around 51 years and normal body shape. In addition, the chief complaints in the two groups were similar. More than half of patients came to the hospital due to abdominal pain, diarrhea, constipation, or bloody stools.

**TABLE 1:** General characteristics

Characteristics	Control group		AI group		*p*
Endoscopist related					
Endoscopist experience					0.447
Naive	14 (1.2%)		15 (1.3%)		
Junior	452 (38.5%)		454 (38.6%)		
Intermediate	377 (32.1%)		346 (29.4%)		
Senior	332 (28.3%)		362 (30.8%)		
Endoscopist gender					0.289
Male	949 (80.8%)		930 (79.0%)		
Female	226 (19.2%)		247 (21.0%)		
Procedure related					
Anesthesia					0.605
Yes	375 (31.9%)		364 (30.9%)		
No	800 (68.1%)		813 (69.1%)		
BBPS score	7.3 ± 1.0		7.3 ± 1.0		0.795
Insertion time (min)	6.4 ± 3.7		6.6 ± 3.9		0.212
Withdrawal time^a^ (min)	7.0 ± 1.8		7.2 ± 1.9		0.121
Patient related					
Patient gender					0.773
Male	595 (50.6%)		603 (51.2%)		
Female	580 (49.4%)		574 (48.8%)		
Patient age (year)	51.7 ± 13.1		50.9 ± 13.5		0.178
Patient BMI (kg/m^2^)	23.0 ± 3.2		22.8 ± 3.2		0.187
Patient waist circumference (cm)	79.6 ± 9.5		79.5 ± 9.3		0.670
Chief complaint					0.468
Abdominal pain	274 (23.3%)		266 (22.6%)		
Diarrhea	164 (14.0%)		154 (13.1%)		
Constipation	96 (8.2%)		94 (8.0%)		
Bloody stools	64 (5.4%)		80 (6.8%)		
Mixed symptoms^b^	38 (3.2%)		54 (4.6%)		
Other symptoms^c^	433 (36.9%)		434 (36.9%)		
Asymptomatic	106 (9.0%)		95 (8.1%)		

Abbreviations: AI, artificial intelligence; BBPS, Boston bowel preparation scale; BMI, body mass index.

^a^ Withdrawal time excludes the time for biopsy.

^b^ Mixed symptoms include abdominal pain and diarrhea, abdominal pain and constipation, abdominal pain and bloody stools, abdominal pain, diarrhea and bloody stools, sometimes diarrhea and sometimes constipation, diarrhea and bloody stools, as well as constipation and bloody stools.

^c^ Other symptoms include changes in stool characteristics, increased stool frequency, bloating, abdominal discomfort, increased anal exhaust, weight loss, abnormalities found by health examination, etc.

### 3.1 Polyp detection outcomes

As shown in Table 2, there were 425 positive patients (patients with at least one polyp detected during colonoscopy) in control group and 457 in AI group. For the primary outcome, the overall PDR of control group and AI group were 36.2% and 38.8%, respectively, and there was no significant difference between two groups (*p* = 0.183).

**TABLE 2:** Primary outcome measure of the two groups

	Control group	AI group	*p*
Positive patients^a^	425	457	
PDR	36.2%	38.8%	0.183

Abbreviations: AI, artificial intelligence; PDR, polyp detection rate.

^a^ Positive patients represent patients with at least one polyp detected during colonoscopy.

The secondary outcome measures are shown in Table 3. A total of 930 polyps were detected in control group, and 1042 polyps were detected in AI group. Among them, there were 505 non‐first polyps (polyps detected after the first one during colonoscopy) in control group and 585 in AI group. For secondary outcomes, the PPP (2.3 vs. 2.2, *p* = 0.113) and PPC (0.9 vs. 0.8, *p* = 0.092) showed no statistical difference between the control and AI groups, while the PPC‐Plus of AI‐assisted colonoscopy was significantly higher than conventional colonoscopy (0.5 vs. 0.4, *p* < 0.05).

**TABLE 3:** Secondary outcome measures of the two groups

	Control group	AI group	*p*
Total polyps	930	1042	
Non‐first polyps^a^	505	585	
PPP	2.2	2.3	0.113
PPC	0.8	0.9	0.092
PPC‐Plus	0.4	0.5	0.024*

Abbreviations: AI, artificial intelligence; PPC, polyps per colonoscopy; PPC‐Plus, non‐first polyps per colonoscopy; PPP, polyps per positive patient.

^a^ Non‐first polyps represent polyps detected after the first one during colonoscopy.

* *p* < 0.05.

### 3.2 Characteristics of detected polyps

The endoscopic features of the detected polyps were analyzed and are shown in Table 4. The distribution of the polyps was similar between the AI and control groups (right colon, 17.9% vs. 16.6%; transverse colon, 17.7% vs. 18.9%; left colon, 64.5% vs. 64.5%. *p* = 0.635), with the majority located in the left colon. For polyp size, compared with conventional colonoscopy, polyps detected by AI‐assisted colonoscopy were more likely to be diminutive polyps (76.0% vs. 68.8%) and less likely to be small polyps (15.1% vs. 21.4%) (*p* < 0.01). In addition, the AI‐assisted colonoscopy detected relatively more flat polyps (5.9% vs. 3.3%) and fewer type Is polyps (61.2% vs. 66.9%) (*p* < 0.05).

**TABLE 4:** Endoscopic features of polyps detected in the two groups

Characteristics	Control group	AI group	*p*
Polyp location			0.635
Right colon	154 (16.6%)	186 (17.9%)	
Transverse colon	176 (18.9%)	184 (17.7%)	
Left colon	600 (64.5%)	672 (64.5%)	
Polyp size			0.004**
Diminutive	476 (68.8%)	624 (76.0%)	
Small	148 (21.4%)	124 (15.1%)	
Large	68 (9.8%)	73 (8.9%)	
Unknown	238	221	
Polyp morphology^a^			0.012*
Ip	56 (6.5%)	78 (7.9%)	
Isp	200 (23.3%)	245 (24.9%)	
Is	574 (66.9%)	601 (61.2%)	
Flat	28 (3.3%)	58 (5.9%)	
Unknown	72	60	

Abbreviations: AI, artificial intelligence.

^a^ Polyp morphology was classified based on the Paris classification scheme: Ip, Isp, Is, and flat polyps (including IIa, IIb, and IIc).

* *p* < 0.05.

** *p* < 0.01.

During the study period, we obtained biopsy results of 430 colorectal polyps, including 227 in control group and 203 in AI group (Table S1). The histological composition of polyps detected by AI‐assisted colonoscopy was similar to that of conventional colonoscopy (*p* = 0.974), most of which were adenomatous polyps, followed by hyperplastic polyps and inflammatory polyps.

### 3.3 Subanalysis by center

As the overall PDR was not significantly improved by AI assistance, we subanalyzed the results by center. Generally, the six centers differed in the experience (naive [<100 colonoscopies], junior [between 100 and 1000 colonoscopies], intermediate [between 1000 and 10,000 colonoscopies], or senior gastroenterologist [>10,000 colonoscopies], which were defined similar to the definitions in Wang’s study^11^) and gender of the endoscopist, examination period (morning or afternoon), BBPS score, insertion, and withdrawal time, as well as patient’s age, BMI, and waist circumference (Table S2).

In ZJU Ningbo, the center with more junior and male endoscopists, as well as more morning examinations, the PDR of AI group was significantly higher than that of control group (50.7% vs. 42.5%, *p* < 0.05), as was PPC (1.2 vs. 1.0, *p* < 0.05) and PPC‐Plus (0.7 vs. 0.6, *p* < 0.05). In Yuyao and Sanmen, where the endoscopists were experienced, procedure time was longer, and patients were slimmer, the PPP and PPC‐Plus of AI group were significantly higher than those of control group. In Taizhou, NBU Ningbo and Yinzhou, there was no significant difference between the AI and control groups. Among them, the PDR in Yinzhou increased by 5.8% but failed to reach a significant level due to limited participants, while for NBU Ningbo, the center with fewer junior and male endoscopists, lower BBPS scores, and older patients, the PDR showed the opposite trend.

**FIGURE 3:**
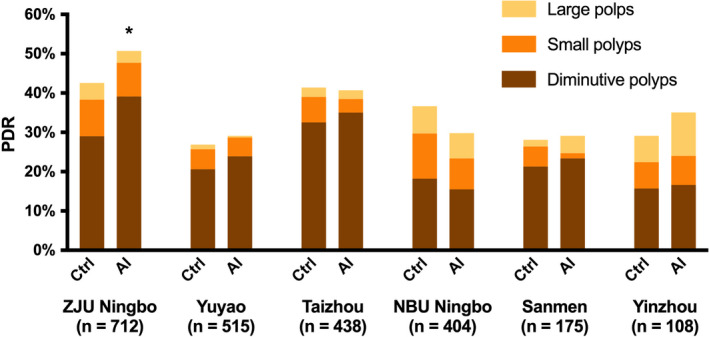
The PDR and polyp size distribution of the control and AI groups in each center. In most centers, the detection of diminutive polyps in AI group showed an increasing trend, and in ZJU Ningbo, the only center with significant improved PDR, there was a significant increase in diminutive polyp detection. *The PDR of control group was significantly higher than that of AI group in ZJU Ningbo center (*p* < 0.05). AI, artificial intelligence; PDR, polyp detection rate

Furthermore, as AI showed advantages in detecting diminutive and flat polyps, we performed a subanalysis of the polyp size and morphology by center. As shown in Figure 3, the detection of diminutive polyps in most centers showed an improving trend, especially in ZJU Ningbo, the only center with significant increased PDR, was also the only one with a significant increase in diminutive polyp detection. However, due to the limited number of flat polyps detected at each center, the polyp morphology did not show significant differences between the two groups.

### 3.4 Factors affecting AI assistance

As the added value of AI assistance varied somewhat between centers, logistic regression analysis was performed to analyze the above factors that may affect polyp detection, including polyp detection method, the experience and gender of the endoscopist, examination period, BBPS score, insertion, and withdrawal time, as well as patient’s age, BMI, and waist circumference (as shown in Table 5).

**TABLE 5:** Logistic regression analysis of factors that may affect PDR

Factors	OR	95% CI	*p*
Polyp detection method	1.248	1.000–1.558	0.049*
Endoscopist experience			0.442
Endoscopist experience (junior)	1.688	0.671–4.242	
Endoscopist experience (intermediate)	1.667	0.663–4.194	
Endoscopist experience (senior)	1.356	0.515–3.572	
Endoscopist gender	1.873	1.404–2.498	<0.001**
Examination period	0.858	0.680–1.082	0.195
BBPS score	0.974	0.868–1.093	0.652
Insertion time	0.966	0.936–0.996	0.027**
Withdrawal time	1.307	1.210–1.411	<0.001**
Patient age	1.046	1.037–1.056	<0.001**
Patient BMI	1.021	0.976–1.067	0.365
Patient waist circumference	1.028	1.012–1.044	<0.001**

Abbreviations: BBPS, Boston bowel preparation scale; BMI, body mass index; CI, confidence interval; OR, odds ratio; PDR, polyp detection rate.

* *p* < 0.05.

** *p* < 0.01.

Among these factors, the polyp detection method independently affected PDR (*p* < 0.05), indicating that AI assistance contributed to the increment of PDR (odds ratio [OR], 1.248; 95% confidence interval, 1.000–1.558). Not only that, endoscopist’s gender (OR, 1.873; *p* < 0.01), insertion (OR, 0.966; *p* < 0.05), and withdrawal time (OR, 1.307; *p* < 0.01), as well as the age (OR, 1.046; *p* < 0.01) and waist circumference of the patient (OR, 1.028; *p* < 0.01) also affected PDR, making the effect of AI more pronounced when the endoscopist was male, the insertion time was shorter but withdrawal time was longer, and the patients were elderly with lager waist circumference. There was no independent impact of endoscopist experience, examination period, BBPS score, and patient BMI on PDR.

In addition, we analyzed these influencing factors of NBU Ningbo center, which was an outlier with the opposite trend in PDR. At this center, although the insertion and withdrawal time, and the patient waist circumference between the two groups were similar, control group seemed to have more elderly patients (54.8 vs. 52.3, *p* = 0.066) and male endoscopists (55.9% vs. 49.7%, *p* = 0.218) than AI group, which may limit or even changeover the AI‐assisted effect to some extent.

## 4 DISCUSSION

In this multicenter RCT, AI group did not show significant increment in overall PDR, but the PPC‐Plus was significantly higher. AI‐assisted colonoscopy detected more diminutive polyps and flat polyps than conventional colonoscopy. No adverse events occurred during the study. In general, real‐time use of AI‐assisted polyp detection system plays a limited role in overall polyp detection, but increases the detection of easily missed polyps, including non‐first polyps, diminutive and flat polyps.

In recent years, there have been a few clinical trials of real‐time AI‐assisted polyp detection,^11^, ^12^, ^13^, ^14^, ^15^ all of which showed significant improvements in polyp and adenoma detection compared with controls (PDR of AI vs. Ctrl, 45.4% [41.1%–49.8%] vs. 30.6% [26.5%–34.6%]; ADR of AI vs. Ctrl, 29.6% [22.2%–37.0%] vs. 19.3% [12.7%–25.9%]).^26^, ^27^ However, the overall PDR in our study did not improve as significantly in these studies, which may be due to our higher baseline PDR (i.e., PDR of Ctrl, 36.2%) than theirs (30.6%), leading to less space for improvement. At the same time, the larger and more complex populations in our multicenter RCT may cause the varied effects in each center, finally resulting to an insignificant overall effect. But on the other hand, AI‐assisted colonoscopy did detect more non‐first polyps, diminutive polyps, and flat polyps than conventional colonoscopy. As previously reported, the presence of more than two polyps increases the risk of missing polyps,^5^ and smaller or flat polyps are more likely to be missed.^28^ Therefore, our AI system can improve the detection of polyps that are easily missed during colonoscopy.

However, our study still had some limitations. First, we did not evaluate the ADR in this study as we only performed biopsy on some typical polyps. According to the recommendations of clinical practice guidelines, biopsy is not necessary for all polyps found during colonoscopy, because it is clinically insignificant and increases medical costs.^29^ For remedy, we compared the histological results of the biopsy polyps between two groups, and found no bias in polyp histological type, which may indicate that the good performance of AI is not due to increased detection of meaningless polyps (non‐adenomatous polyps). In addition, we will overcome this limitation in future studies and follow up the study population to assess long‐term clinical prognosis. Second, the sensitivity and specificity of the system are slightly lower than some other systems. Last but not least, the multicenter nature of our study could have caused some variation in PDR across centers, thereby compromising the overall performance of the AI system.

Further analysis of potential influencing factors showed that PDR was significantly affected by AI assistance, as well as the endoscopist gender, insertion and removal time, and patient’s waist circumference. These factors varied somewhat between centers and may limit the impact of AI to some extent, resulting in a less significant overall effect. It has been shown that when the withdrawal time is longer,^22^ patients are elderly^23^ or abdominal obese,^25^ the PDR is relatively high, which may be due to sufficient time for inspection and a higher polyp incidence. In our study, under conditions associated with higher PDR, the impact of AI assistance was also greater. In addition, male endoscopists (possibly more compulsive and thorough in personality)^20^ did better than females in AI‐assisted polyp detection. However, other previously reported influencing factors, including endoscopist experience, examination period, BBPS score, and patient BMI,^21^, ^24^, ^30^ did not significantly affect PDR in this study, suggesting that AI is a great help in improving polyp detection and reducing dependence.

In this multicenter RCT, although the improvement in PDR has not reached a significant level, AI‐assisted colonoscopy can effectively detect polyps that are easily missed in conventional colonoscopy, including non‐first polyps, diminutive polyps, and flat polyps. Given the complexity of multi‐centers, we will update the system and develop operating standards for AI‐assisted colonoscopy in future studies to make the most of it in polyp detection.

## CONFLICT OF INTERESTS

The authors declare that they have no conflict of interest.

## Supporting information

Table S1‐2

## Data Availability

The data that support the findings of this study are available from the corresponding author upon reasonable request.
