# Supplementary material for: Artificial intelligence‐assisted colonoscopy: A prospective, multicenter, randomized controlled trial of polyp detection
Source: Cancer Med. 2021 Sep 3;10(20):7184–93. doi: 10.1002/cam4.4261 (PMC8525182; doi:10.1002/cam4.4261)
Supplement: Supplementary file 1 — Table S1‐2 [file CAM4-10-7184-s001.docx]

**Supplemental Table S1.** Histology of biopsy polyps in the two groups

| Histology ^a^ | Control group | AI group | *P* |
| --- | --- | --- | --- |
| Total biopsy polyps | 227 | 203 | 0.974 |
| Adenomatous polyps | 150 (66.1%) | 130 (64.0%) |  |
| Hyperplastic polyps | 39 (17.2%) | 36 (17.7%) |  |
| Inflammatory polyps | 34 (15.0%) | 33 (16.3%) |  |
| Canceration | 4 (1.8%) | 4 (2.0%) |  |

^a^ There were no hamartomatous polyps in the biopsy polyps.

*AI*, artificial intelligence.

**Supplemental Table S2.** Subanalysis of the characteristics of each center

| Characteristics | ZJU Ningbo | Yuyao | Taizhou | NBU Ningbo | Sanmen | Yinzhou | *P* |
| --- | --- | --- | --- | --- | --- | --- | --- |
| Endoscopist experience ^a^ |  |  |  |  |  |  | < 0.001** |
| Naive | 0 (0.0%) | 0 (0.0%) | 36 (8.0%) | 0 (0.0%) | 0 (0.0%) | 0 (0.0%) |  |
| Junior | 538 (75.6%) | 30 (5.8%) | 308 (68.8%) | 0 (0.0%) | 0 (0.0%) | 30 (27.8%) |  |
| Intermediate | 174 (24.4%) | 66 (12.8%) | 97 (21.7%) | 235 (58.2%) | 132 (76.7%) | 19 (17.6%) |  |
| Senior | 0 (0.0%) | 419 (81.4%) | 7 (1.6%) | 169 (41.8%) | 40 (23.3%) | 59 (54.6%) |  |
| Endoscopist gender |  |  |  |  |  |  | < 0.001** |
| Male | 700 (98.3%) | 503 (97.7%) | 302 (68.9%) | 203 (50.2%) | 106 (61.6%) | 65 (60.2%) |  |
| Female | 12 (1.7%) | 12 (2.3%) | 136 (31.1%) | 201 (49.8%) | 66 (38.4%) | 43 (39.8%) |  |
| Examination period |  |  |  |  |  |  | < 0.001** |
| Morning | 440 (61.8%) | 21 (4.1%) | 132 (30.1%) | 207 (51.2%) | 0 (0.0%) | 0 (0.0%) |  |
| Afternoon | 272 (38.2%) | 494 (95.9%) | 306 (69.9%) | 197 (48.8%) | 175 (100.0%) | 108 (100.0%) |  |
| BBPS score | 7.4 ± 1.0 | 7.2 ± 1.0 | 7.4 ± 0.9 | 6.9 ± 0.9 | 7.0 ± 0.9 | 8.0 ± 1.0 | < 0.001** |
| Insertion time (min) | 7.2 ± 4.8 | 5.7 ± 2.5 | 6.4 ± 2.6 | 5.0 ± 2.8 | 10.8 ± 3.9 | 4.8 ± 2.3 | < 0.001** |
| Withdrawal time ^b^ (min) | 6.8 ± 1.4 | 6.5 ± 1.6 | 8.2 ± 1.9 | 6.8 ± 1.7 | 8.4 ± 2.9 | 6.5 ± 0.9 | < 0.001** |
| Patient gender |  |  |  |  |  |  | 0.069 |
| Male | 354 (49.7%) | 279 (55.2%) | 203 (46.3%) | 214 (53.0%) | 93 (53.1%) | 49 (45.4%) |  |
| Female | 358 (50.3%) | 226 (44.8%) | 235 (53.7%) | 190 (47.0%) | 82 (46.9%) | 59 (54.6%) |  |
| Patient age (year) | 51.2 ± 13.7 | / | 49.5 ± 12.7 | 53.6 ± 13.5 | 51.5 ± 12.2 | 50.4 ± 12.6 | < 0.001** |
| Patient BMI (kg/m^2^) | 22.9 ± 3.3 | 22.3 ± 3.0 | 23.2 ± 3.3 | 23.0 ± 3.1 | 23.2 ± 3.3 | 22.6 ± 2.8 | < 0.001** |
| Patient waist circumference (cm) | 80.7 ± 9.2 | 77.4 ± 8.1 | 80.0 ± 10.2 | 80.1 ± 9.2 | 77.5 ± 7.9 | 77.9 ± 12.9 | < 0.001** |

^a^ For endoscopist experience, "naive" refers to the gastroenterology resident with less than 100 colonoscopy experiences; "junior" refers to the attending physician with hundreds of colonoscopy experiences; "intermediate" refers to the associate chief physician with thousands of colonoscopy experience; "senior" refers to the chief physician with tens of thousands of colonoscopy experiences.

^b^ Withdrawal time excludes the time for biopsy.

*BBPS*, Boston bowel preparation scale; *BMI*, body mass index.

**P* < 0.05, ***P* < 0.01
